# Preoperative inflammatory markers predict postoperative clinical outcomes in patients undergoing heart valve surgery: A large-sample retrospective study

**DOI:** 10.3389/fimmu.2023.1159089

**Published:** 2023-03-31

**Authors:** Hongni Tian, Xuetao Jiang, Guangyou Duan, Jie Chen, Qi Liu, Yamei Zhang, Shiqi Li, Xiaohang Bao, He Huang

**Affiliations:** ^1^ Department of Anesthesiology, The Second Affiliated Hospital, Chongqing Medical University, Chongqing, China; ^2^ Department of Anesthesiology, Second Affiliated Hospital of Army Military Medical University, Chongqing, China

**Keywords:** valvular cardiac surgery, inflammation, postoperative outcomes, C-reactive protein, neutrophil-to-lymphocyte ratio

## Abstract

**Introduction:**

Preoperative inflammation affects the postoperative outcomes of patients undergoing heart valve surgery. This study aimed to explore the role and predictive effects of preoperative inflammation on the primary outcomes after valvular cardiac surgery.

**Methods:**

This retrospective study utilized a medical recording system to screen 5075 patients who underwent heart valve surgery. Data on the C-reactive protein (CRP) levels, erythrocyte sedimentation rate (ESR), and neutrophil-to-lymphocyte ratio (NLR) before heart valve surgery were collected from the hospital’s medical system. Postoperative hepatic insufficiency, acute kidney injury, heart failure, and myocardial damage were assessed using blood indicators. Patients with and without prolonged mechanical ventilation, extended intensive care unit stays, prolonged hospital stays, and death within 30 days after surgery (considered the primary outcome in this study) were compared. Group comparisons, receiver operating characteristic (ROC) curve analyses, and logistic analyses were performed to determine the associations between preoperative inflammation and outcomes after heart valve surgery.

**Results:**

A total of 3249 patients were included in the analysis. Significant differences in CRP level, ESR, and NLR were found between patients with and without postoperative adverse outcomes. ROC analysis showed that CRP levels >5 mg/L effectively predicted postoperative heart failure, and NLR >3.5 had a good predictive effect on all-cause mortality within 30 days after surgery. Patients with CRP levels >5 mg/L had a higher incidence of postoperative heart failure than other patients (20.7% vs. 12.6%, *P*<0.001), with a relative risk of 1.447 (95% confidence interval: 1.155–1.814). Patients with NLR >3.5 had a higher incidence of death within 30 days after surgery (5.3% vs. 1.2%, *P*<0.001), with a relative risk of 3.236 (95% confidence interval: 1.773–5.906).

**Conclusion:**

Preoperative inflammation can affect postoperative outcomes in patients undergoing heart valve surgery. CRP level >5 mg/L and NLR >3.5 can effectively predict postoperative heart failure and death within 30 days after surgery, respectively.

## 1 Introduction

Inflammation is an important pathogenic factor in valvular diseases (1, 2). Surgery is one of the main treatments for valvular disease, and the number of heart valve operations is increasing (3). However, the incidence of organ function injury and related complications after surgery remains high (4). Early prediction of the risk of postoperative complications and mortality is important for the improved treatment of patients undergoing heart valve surgery. Although few studies have attempted to predict thromboembolism or neurobehavioral outcomes using preoperative blood biochemical indicators (5, 6), studies on the association between preoperative blood biochemical indicators and other postoperative complications and mortality are insufficient. A large number of studies have shown that the preoperative inflammatory state of patients undergoing cardiovascular surgery is closely related to the incidence and all-cause mortality of postoperative complications, including bleeding, deep wound infection, and mortality (7–12). Currently, the main method to assess an inflammatory state is the testing of blood biomarkers, which is easy to access and apply. C-reactive protein (CRP), neutrophil-to-lymphocyte ratio (NLR), and erythrocyte sedimentation rate (ESR) are commonly used objective inflammatory biomarkers. Previous studies have demonstrated their predictive effects on the outcomes of aneurysmal subarachnoid hemorrhage (13), cancer (14), and perioperative myocardial injury in non-cardiac surgery patients (15). However, to date, the prognostic role of these preoperative inflammatory markers in heart valve surgery under cardiopulmonary bypass (CBP) has not been synthetically investigated, highlighting a need for research on the topic.

Therefore, this study aimed to retrospectively collect preoperative and postoperative clinical data of patients undergoing heart valve surgery and to evaluate and compare the predictive ability of preoperative inflammatory states determined by CRP levels, ESR, and NLR in terms of different clinical outcomes, including organ dysfunction and 30-day mortality, in patients undergoing heart valve surgery under CBP. Additionally, this study aimed to determine the optimal predictive value for use in clinical practice.  

## 2 Materials and methods

### 2.1 Patients and ethics

This was a single-center retrospective cohort study. The study protocol was approved by the Ethics Committee of the Second Affiliated Hospital of the Army Medical University (No. 2022-457-01) on Oct 17, 2022. The study was conducted in accordance with the Declaration of Helsinki, and the confidentiality of patient data was guaranteed. Because of the study’s retrospective nature, the informed consent requirement was waived. This study included patients who underwent heart valve surgery at the Second Affiliated Hospital of Army Medical University between October 2016 and December 2020. The inclusion criteria were patients undergoing heart valve replacement or repair under CPB, including those requiring concurrent coronary artery bypass grafting, atrial/ventricular septal defect repair, or modified maze surgery (in which atria are incised and successively sutured in a maze route to relieve atrial fibrillation). Patients who were under 18 years of age, in an emergency state before surgery, underwent transcatheter valve replacement, underwent aortic surgery, or lacked data needed for laboratory tests or follow-up to assess inflammatory status were excluded.

### 2.2 Data collection

The patient data used in this study were collected from the electronic medical record system. The collected data included demographic information such as sex, age, height, weight, body mass index (BMI), preoperative complications, smoking and alcohol consumption, American Society of Anesthesiologists grading, New York Heart Association grading of cardiac function, and laboratory test data, including inflammation-related indicators (CRP, NLR, and ESR). All preoperative examinations and test results were collected within 3 days prior to the operation; if a patient had multiple preoperative examinations, the results from the examination closest to the operation day were included.

CRP is one of the most important acute-phase proteins and a sensitive biomarker of inflammation and tissue damage (16). ESR is a widely used laboratory indicator in clinical practice and is significantly elevated in chronic diseases characterized by tissue damage and inflammation (17). NLR is the ratio of the neutrophil count to the lymphocyte count in routine blood tests, which can comprehensively reflect the increase in neutrophils and decrease in lymphocytes in the inflammatory state (18).

Additional data included echocardiography results (e.g., presence or absence of atrial thrombus), surgery types (including single valve surgery, combined multi-valve surgery, coronary artery bypass grafting with valve surgery, and simple atrial/ventricular septal defect repair with valve surgery), etiological diagnosis (rheumatic, degenerative, congenital, and infective endocarditis) and morphological diagnosis (simple stenosis, simple insufficiency, and stenosis with insufficiency), and postoperative markers of liver and kidney function, heart failure, and myocardial infarction.

### 2.3 Clinical outcomes

In this study, the primary outcome was all-cause mortality at 30 days after surgery. Other clinical outcomes included invasive ventilator support time, intensive care unit (ICU) stay time, postoperative hospital stay, and impairment of postoperative organ function, including acute kidney injury (AKI) (19). AKI was defined as any of the following: increase in serum creatinine (SCr) by ≥0.3 mg/dl (≥26.5 μmol/L) within 48 h; an increase in SCr to ≥1.5 times from baseline, which is known or presumed to have occurred within the prior 7 days; and urine volume <0.5 ml/kg/h for 6 h. Hepatic insufficiency (20) was defined as bilirubin levels >50 µmol/L at any time point between postoperative day 1 and postoperative day 5. Postoperative cardiac dysfunction after cardiac surgery (21) was defined as cardiac troponin I (cTnI) level >5.4 ng/ml, and heart failure was defined as brain natriuretic peptide (BNP) level >400 pg/ml within 24 h. In addition, prolonged mechanical ventilation was defined as postoperative mechanical ventilation time > 24 h (22). Extended ICU stay was defined as a postoperative ICU stay ≥5 days (23). Prolonged hospital stay was defined as a postoperative length of stay >3 weeks (24).

### 2.4 Statistical analysis

SPSS26.0 (IBM Corp., Armonk, NY, USA) was used for statistical analysis, with bilateral *P*<0.05 being considered statistically significant. Mean ± standard deviation was used to represent continuous variables of the overall distribution, median (interquartile spacing) was used to represent non-normal distribution data, and quantity (percentage) was used to represent qualitative variables. First, patients were grouped according to the occurrence of different clinical outcome variables, and differences in inflammatory indicators (CRP level, ESR, and NLR) among the different groups were compared. Because all inflammatory indicators were abnormally distributed, the Mann–Whitney U test was used for comparison. Second, the receiver operating characteristic (ROC) curve was used to explore the predictive abilities of these different inflammatory indicators in distinguishing the early postoperative outcome of patients undergoing heart valve surgery, and the optimal cut-off point was calculated based on the Youden index score. Finally, the optimal cut-off value was used to group the patients according to the level of inflammation, and univariate logistic regression was used to explore the predictive efficacy of different preoperative factors for major clinical outcomes. Factors with a significance level of *P*<0.05 were included in the multivariate logistic regression analysis to further verify the predictive value of the inflammation index for postoperative outcomes. The relative risk (RR) with the corresponding 95% confidence interval was calculated for different indicators.

## 3 Results

**Figure 1:**
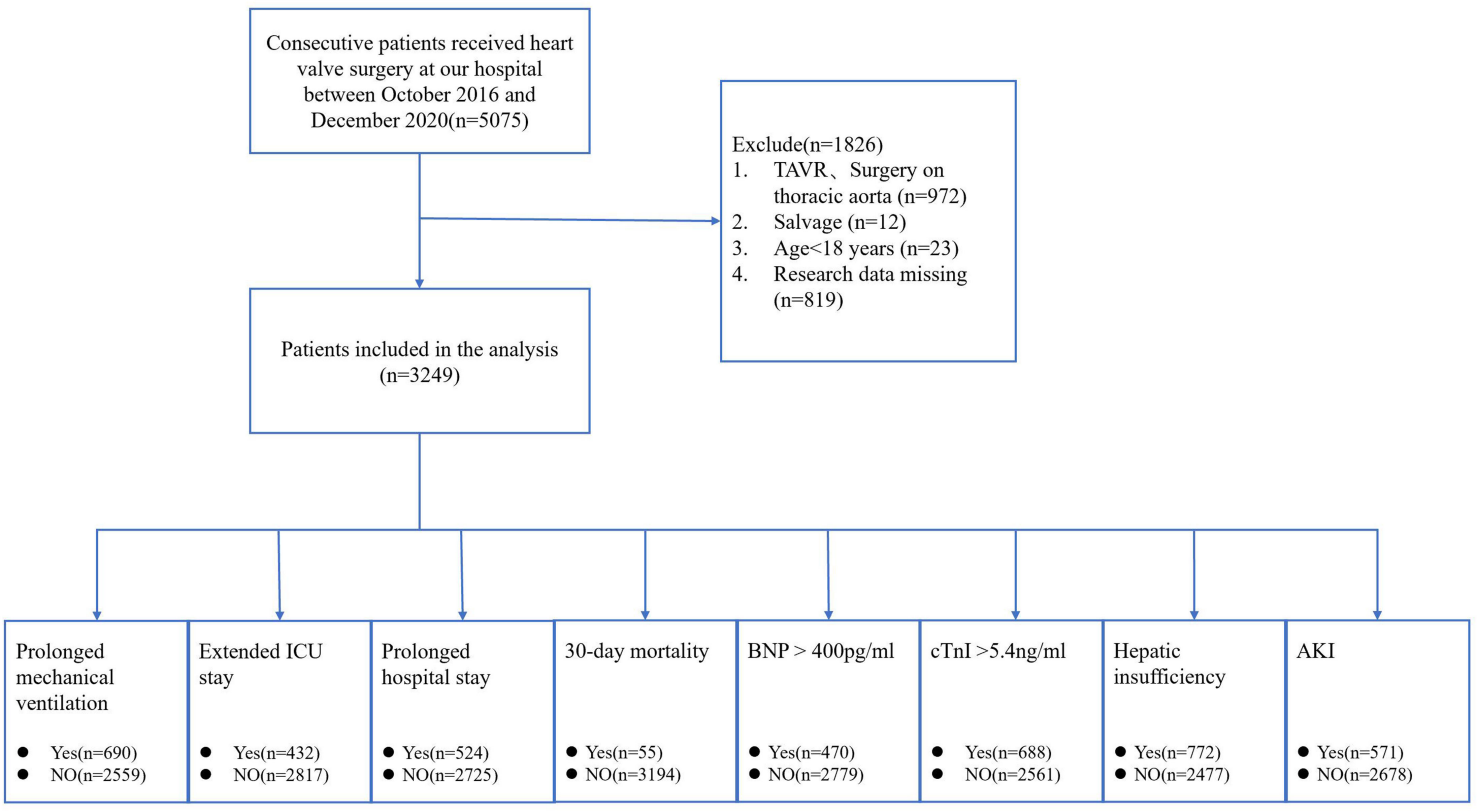
Flow chart of the study. TAVR, transcatheter aortic valve replacement; ICU, intensive care unit; BNP, brain natriuretic peptide; cTnI, cardiac troponin I; AKI, acute kidney injury.

A total of 5075 patients who underwent heart valve surgery were identified from the medical record system. Among these patients, 972 underwent transcatheter aortic valve replacement or combined surgery on the thoracic aorta, 12 were salvaged (patients requiring cardiopulmonary resuscitation en route to the operating theatre or prior to induction of anesthesia), 23 were aged <18 years, and 819 were excluded because of missing data. Finally, 3249 patients were included in the analysis (**Figure 1**). The demographic and clinical data of all the included patients are presented in **Table 1**. For all included patients, the postoperative 30-day mortality was 1.69% (55 patients), 21.24% (690 patients) presented prolonged mechanical ventilation, 13.30% (432 patients) had an extended ICU stay, 16.13% (524 patients) had a prolonged hospital stay, 14.47% (470 patients) presented with a BNP level >400 pg/ml, 21.18% (688 patients) presented with a cTnI level >5.4 ng/ml, 23.76% (772 patients) presented with hepatic insufficiency, and 17.57% (571 patients) presented with postoperative AKI.

**Table 1:** Demographic and clinical data.

Variable	Total (N=3249)
Gender (Female), n (%)	61.65%
Age, years	51.84 ± 9.44
Age group, n (%)	
≤45 years	684 (21.05%)
45 to 60 years	1898 (58.42%)
≥60 years	667 (20.53%)
Height, cm	158.87 ± 8.23
Weight, kg	58.91 ± 9.72
BMI, kg/m^2^	23.30 ± 3.17
BMI group, n (%)	
≤18.5 kg/m^2^	167 (5.14%)
18.5 to 24 kg/m^2^	1831 (56.36%)
≥24 kg/m^2^	1251 (38.50%)
Smoking history, n (%)	666 (20.50%)
Drinking history, n (%)	532 (16.37%)
Coronary heart disease, n (%)	316 (9.73%)
Hypertension, n (%)	334 (10.28%)
Diabetes mellitus, n (%)	129 (3.97%)
Renal insufficiency, n (%)	142 (4.37%)
Hepatic insufficiency, n (%)	395 (12.16%)
Stroke, n (%)	214 (6.59%)
Atrial thrombosis, n (%)	365 (11.23%)
Arrhythmia, n (%)	1682 (51.77%)
ASA grade, n (%)	
II	30 (0.01%)
III	2427 (74.7%)
IV	792 (24.38%)
NYHA class, n (%)	
II	749 (23.05%)
III	2143 (65.96%)
IV	357 (10.99%)
Etiological diagnosis of valves, n (%)	
Rheumatic	2430 (74.79%)
Congenital	119 (3.66%)
Degenerative	591 (18.19%)
Infective Endocarditis	109 (3.35%)
Morphological diagnosis of valves, n (%)	
insufficiency	733 (22.56%)
stenosis	211 (6.49%)
Insufficiency + stenosis	2305 (70.94%)
Surgical approaches, n (%)	
Isolated valve surgery	936 (28.81%)
Combined valve surgery	2167 (66.70%)
Valve + CABG/heart septal defects	146 (4.49%)
Maze Procedure, n (%)	746 (22.96%)
Automatic heartbeat recovery, n (%)	2641 (81.29%)
Operative time, min	231.02 ± 58.51
CPB time, min	118.30 ± 40.40
ACC time, min	78.12 ± 30.49

BMI, Body mass index; ASA, American Society of Anesthesiologists; NYHA, New York Heart Association; CABG, coronary artery bypass graft; CPB, cardiopulmonary bypass; ACC, aortic cross-clamp.

**Figure 2:**
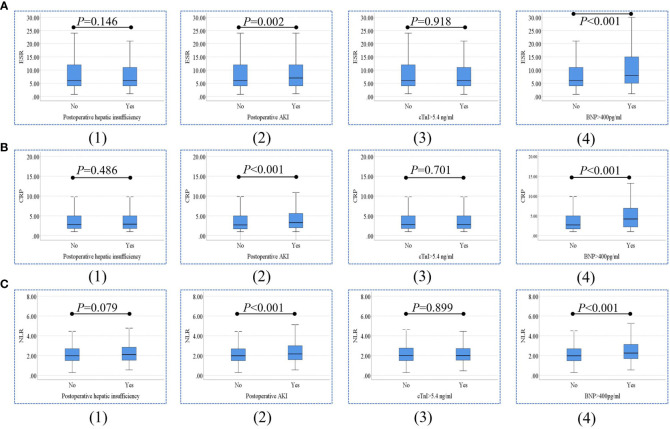
Comparisons of ESR **(A)**, CRP levels **(B)**, and NLR **(C)** between patients with and without postoperative hepatic insufficiency (1), AKI (2), cTnI level >5.4 ng/ml (3), and BNP level >400 pg/ml (4). ESR, erythrocyte sedimentation rate; CRP, C-reactive protein; NLR, neutrophil-to-lymphocyte ratio; BNP, brain natriuretic peptide; cTnI, cardiac troponin I; AKI, acute kidney injury.

Comparisons of ESR, CRP, and NLR in patients with and without postoperative hepatic insufficiency, AKI, cTnI level >5.4 ng/ml, and BNP level >400 pg/ml are shown in **Figure 2**. ESR was significantly higher in patients with postoperative AKI (*P*=0.002) and BNP level >400 pg/ml (*P*<0.001) than in other patients. Both CRP levels and NLR were higher in patients with postoperative AKI (*P*<0.001) and BNP level >400 pg/ml (*P*<0.001) than in other patients.

**Figure 3:**
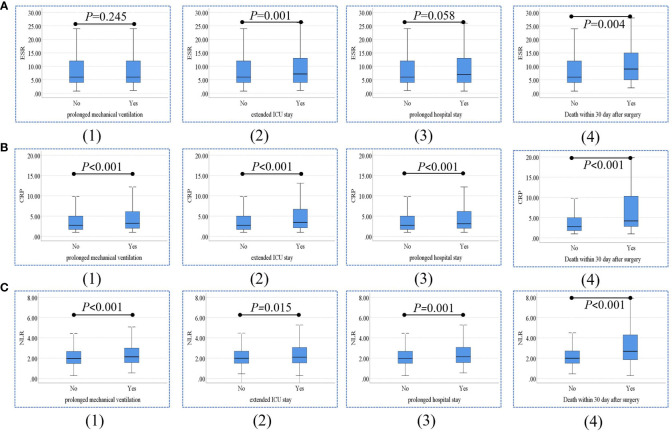
Comparisons of ESR **(A)**, CRP levels **(B)**, and NLR **(C)** between patients with and without prolonged mechanical ventilation (1), extended ICU stay (2), prolonged hospital stay (3), and death within 30 days after surgery (4). ESR, erythrocyte sedimentation rate; CRP, C-reactive protein; NLR, neutrophil-to-lymphocyte ratio; ICU, intensive care unit.

As shown in **Figure 3**, the ESR, CRP level, and NLR in patients with and without prolonged mechanical ventilation extended ICU stay, prolonged hospital stay, and death within 30 days after surgery were compared. ESR level was significantly higher in patients with an extended postoperative ICU stay (*P*=0.001) and death within 30 days after surgery (*P*=0.004) than in other patients. CRP levels in patients with prolonged mechanical ventilation (*P*<0.001), extended ICU stay (*P*<0.001), prolonged hospital stay (*P*<0.001), and death within 30 days after surgery (*P*<0.001) were significantly higher than those in other patients. Moreover, NLR levels were significantly higher in patients with prolonged mechanical ventilation (*P*<0.001), extended ICU stay (*P*=0.015), prolonged hospital stay (*P*=0.001), and death within 30 days (*P*<0.001).

**Figure 4:**
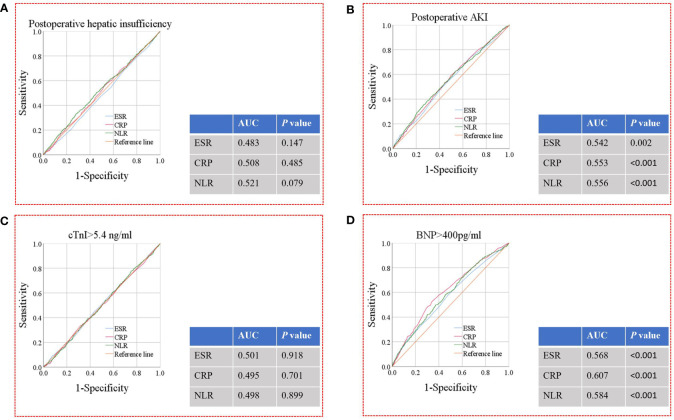
ROC analysis for ESR, CRP levels and NLR for the prediction of postoperative hepatic insufficiency **(A)**, AKI **(B)**, cTnI level >5.4 ng/ml **(C)**, and BNP level >400 pg/ml **(D)**. ROC, receiver operating characteristic; AKI, acute kidney injury; ESR, erythrocyte sedimentation rate; CRP, C-reactive protein; NLR, neutrophil-to-lymphocyte ratio; AUC, area under the curve.

The results of the ROC analysis for ESR, CRP, and NLR in predicting postoperative hepatic insufficiency, AKI, cTnI level >5.4 ng/ml, and BNP level >400 pg/ml are shown in **Figure 4**. ESR (area under the curve [AUC]=0.542, *P*=0.002), CRP level (AUC=0.553, *P*<0.001), and NLR (AUC=0.556, *P*<0.001) had a significant predictive effect on postoperative AKI. In addition, ESR (AUC=0.568, *P*<0.001), CRP level (AUC=0.607, *P*<0.001), and NLR (AUC=0.584, *P*<0.001) significantly predicted the occurrence of postoperative BNP level >400 pg/ml. Based on the above ROC analysis, we calculated an optimal CRP cut-off value of 5 mg/L for predicting a postoperative BNP level >400 pg/ml according to the Youden Index.

**Figure 5:**
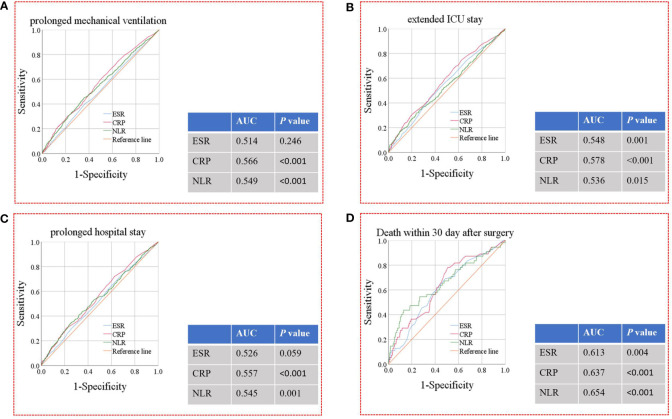
ROC analysis for ESR, CRP levels and NLR for the prediction of prolonged mechanical ventilation **(A)**, extended ICU stay **(B)**, prolonged hospital stay **(C)**, and death within 30 days after surgery **(D)**. ROC, receiver operating characteristic; ESR, erythrocyte sedimentation rate; CRP, Creactive protein; NLR, neutrophil-to-lymphocyte ratio; ICU, intensive care unit; AUC, area under the curve.

**Figure 5** shows the results of the ROC analysis of ESR, CRP, and NLR in predicting postoperative prolonged mechanical ventilation, extended ICU stay, prolonged hospital stay, and death within 30 days after surgery. CRP level (AUC=0.566, *P*<0.001) and NLR (AUC=0.549, *P*<0.001) had a significant predictive effect on prolonged mechanical ventilation. ESR (AUC=0.548, *P*=0.001), CRP level (AUC=0.578, *P*<0.001), and NLR (AUC=0.536, *P*=0.015) had significant predictive effects on extended ICU stay. CRP level (AUC=0.557, *P*<0.001) and NLR (AUC=0.545, *P*=0.001) had a significant predictive effect on prolonged hospital stay. In addition, ESR (AUC=0.613, *P*=0.004), CRP level (AUC=0.637, *P*<0.001), and NLR (AUC=0.654, *P*<0.001) had significant predictive effects on death within 30 days after surgery. Based on the above ROC analysis, we calculated an optimal NLR cut-off value of 3.5 for predicting death within 30 days after surgery, according to the Youden Index.

The results of the univariate analysis for postoperative BNP level >400 pg/ml are listed in **Table 2**. The age group (*P*=0.011), BMI group (*P*=0.033), renal insufficiency (*P*<0.001), atrial thrombosis (*P*=0.012), NYHA class (*P*<0.001), etiological diagnosis of valves (*P*=0.002), surgical approaches (*P*<0.001), maze procedure (*P*<0.001), operative time (*P*<0.001), CPB time (*P*<0.001), aortic cross-clamp (ACC) time (*P*<0.001), and CRP group (*P*<0.001) were identified as significant risk factors for postoperative BNP level >400 pg/ml. In the multivariable logistic regression analysis (**Table 3**), CRP group (*P*=0.001) with an RR=1.447 (95% confidence interval [95% CI]: 1.155–1.814) was also identified as an independent factor for postoperative BNP level >400 pg/ml. The incidence of postoperative BNP level >400 pg/ml in patients with CRP level >5 mg/L was significantly higher than that in patients with CRP level ≤5 mg/L (20.7% *vs.* 12.6%, *P*<0.001).

**Table 2:** Univariable analysis for postoperative BNP level >400 pg/ml.

Variables	Wald *x* ^2^	*P* value
Gender	0.001	0.980
Age group	6.474	0.011
BMI group	4.571	0.033
Smoking history	0.894	0.344
Drinking history	0.070	0.792
Coronary heart disease	1.939	0.164
Hypertension	0.591	0.442
Diabetes mellitus	0.180	0.672
Renal insufficiency	32.727	<0.001
Hepatic insufficiency	1.823	0.177
Stroke	0.374	0.541
Atrial thrombosis	6.371	0.012
Arrhythmia	0.110	0.741
ASA grade	3.526	0.060
NYHA class	73.972	<0.001
Etiological diagnosis of valves	10.010	0.002
Morphological diagnosis of valves	2.696	0.101
Surgical approaches	28.429	<0.001
Maze Procedure	20.749	<0.001
Automatic heartbeat recovery	0.811	0.368
Operative time, min	24.075	<0.001
CPB time, min	27.352	<0.001
ACC time, min	26.030	<0.001
CRP group	30.348	<0.001

BMI, body mass index; ASA, American Society of Anesthesiologists; NYHA, New York Heart Association; CABG, coronary artery bypass graft; CPB, cardiopulmonary bypass; ACC, aortic cross-clamp; CRP, C-reactive protein

**Table 3:** Multivariable logistic regression analysis for postoperative BNP >400 pg/ml.

Variables	Wald *x* ^2^	*P* value	RR	95% CI
Age group	10.593	0.005		
Age ≤45 years (Ref. 45–60)	3.517	0.061	0.764	0.577–1.012
Age ≥60 years (Ref. 45–60)	4.339	0.036	1.306	1.018–1.675
BMI group	30.278	<0.001		
BMI ≤18.5 kg/m^2^ (Ref. 18.5–24)	16840	<0.001	2.209	1.513–3.226
BMI ≥24 kg/m^2^ (Ref. 18.5–24)	7.557	0.006	0.732	0.586–0.914
Renal insufficiency (Ref. No)	13.357	<0.001	2.094	1.409–3.113
NYHA class	56.553	<0.001		
III (Ref. II)	1.685	0.194	1.205	0.909–1.598
IV (Ref. II)	41.427	<0.001	3.132	2.212–4.435
Maze Procedure (Ref. Yes)	25.004	<0.001	2.055	1.550–2.726
Surgical approaches	9.803	0.007		
Combined valve surgery (Ref. Isolated valve surgery)	7.558	0.006	1.449	1.112–1.888
Valve + CABG/heart septal defects (Ref. Isolated valve surgery)	6.688	0.010	1.885	1.166–3.048
ACC time, min	14.939	<0.001	1.007	1.003–1.010
CRP group (Ref. ≤5 mg/L)	10.296	0.001	1.447	1.155–1.814

BMI, body mass index; RR, relative risk; CI, confidence interval; Ref., reference; NYHA, New York Heart Association; CABG, coronary artery bypass graft; ACC, aortic cross-clamp; CRP, C-reactive protein.

The results of the univariate analysis of deaths within 30 days after surgery are shown in **Table 4**. Sex (*P*=0.029), age group (*P*<0.001), BMI group (*P*<0.001), coronary heart disease (*P*<0.001), renal insufficiency (*P*<0.001), atrial thrombosis (*P*=0.006), ASA grade (*P*<0.001), NYHA class (*P*<0.001), surgical approaches (*P*<0.001), operative time (*P*<0.001), CPB time (*P*<0.001), ACC time (*P*<0.001), and NLR group (*P*<0.001) were identified as significant risk factors for death within 30 days after surgery. In the multivariate logistic regression analysis (**Table 5**), NLR group (*P*<0.001) with RR=3.236 (95% CI: 1.773–5.906) was also identified as an independent factor for death within 30 days after surgery. The incidence of death within 30 days after surgery in patients with NLR >3.5 was significantly higher than that of patients with NLR ≤3.5 (5.3% *vs.* 1.2%, *P*<0.001).

**Table 4:** Univariable analysis for death within 30 days after surgery.

Variables	Wald *x* ^2^	*P* value
Gender	4.757	0.029
Age group	24.618	<0.001
BMI group	21.043	<0.001
Smoking history	0.060	0.807
Drinking history	0.136	0.712
Coronary heart disease	14.111	<0.001
Hypertension	1.089	0.297
Diabetes mellitus	0.016	0.898
Renal insufficiency	16.093	<0.001
Hepatic insufficiency	3.129	0.077
Stroke	0.116	0.733
Atrial thrombosis	7.690	0.006
Arrhythmia	2.231	0.135
ASA grade	12.861	<0.001
NYHA class	28.743	<0.001
Etiological diagnosis of valves	0.055	0.814
Morphological diagnosis of valves	0.180	0.672
Surgical approaches	16.216	<0.001
Maze Procedure	0.196	0.658
Automatic heartbeat recovery	1.650	0.199
Operative time, min	44.227	<0.001
CPB time, min	41.027	<0.001
ACC time, min	24.291	<0.001
NLR group	30.542	<0.001

BMI, body mass index; NYHA, New York Heart Association; CRP, C-reactive protein; ASA, American Society of Anesthesiologists; CPB, cardiopulmonary bypass; ACC, aortic cross-clamp; NLR, neutrophil-to-lymphocyte ratio.

**Table 5:** Multivariable logistic regression analysis for death within 30 days after surgery.

Variables	Wald *x* ^2^	*P* value	RR	95% CI
Age group	19.722	<0.001		
Age ≤45 years (Ref.45–60)	2.989	0.084	0.398	0.140–1.131
Age ≥60 years (Ref.45–60)	11.674	0.001	2.843	1.561–5.176
BMI group	15.460	<0.001		
BMI ≤18.5 kg/m^2^ (Ref. 18.5–24)	13.726	<0.001	4.590	2.050–10.279
BMI ≥24 kg/m^2^ (Ref. 18.5–24)	0.045	0.832	0.932	0.486–1.788
NYHA class	11.212	0.004		
III (Ref. II)	1.056	0.304	1.755	0.600–5.128
IV (Ref. II)	6.758	0.009	4.544	1.451–14.226
CPB time, min	31.835	<0.001	1.014	1.009–1.019
NLR group (Ref. ≤3.5)	14.627	<0.001	3.236	1.773–5.906

BMI, body mass index; NYHA, New York Heart Association; CPB, cardiopulmonary bypass; NLR, neutrophil-to-lymphocyte ratio; RR, relative risk; CI, confidence interval.

## 4 Discussion

Differing from most previous studies, the current study mainly focused on patients undergoing heart valve surgery and their preoperative inflammatory index. In addition, the sample size of the study is relatively large, and it synthetically included several different postoperative outcomes. The results showed significant differences in preoperative CRP levels, ESR, and NLR between patients with and without adverse postoperative outcomes. Moreover, the ROC diagnostic analysis showed that CRP (>5 mg/L) and NLR (>3.5) had significant predictive ability for early postoperative cardiac insufficiency and death within 30 days after surgery, respectively. Further multivariate logistic regression analysis revealed that CRP level >5 mg/L was an independent predictor of early postoperative cardiac insufficiency, and NLR >3.5 was an independent predictor of death within 30 days after surgery.

In the first part of the study, patients were grouped according to whether they had adverse postoperative outcomes, and differences in preoperative inflammatory markers were compared between groups. We found statistically significant differences in the preoperative CRP level, ESR, and NLR among patients with and without postoperative AKI and BNP level >400 pg/ml. There were also statistically significant differences in preoperative CRP levels and NLR between patients with and without prolonged mechanical ventilation and hospital stay. Finally, the preoperative CRP level, ESR, and NLR were significantly different among patients with and without an extended ICU stay and death within 30 days after surgery. These findings indicate that a high level of preoperative inflammation may play an important role in various postoperative organ functional injuries and clinical outcomes. A recent retrospective study showed that patients who died 1 year after heart valve surgery had significantly higher preoperative CRP levels than those who survived (4). In another study involving male patients who underwent coronary bypass surgery, an elevated ESR was associated with postoperative AKI, prolonged hospital stay, and heart failure (10). In addition, a retrospective study of cardiac surgery found that NLR ≥3.23 was associated with increased mortality 30 days after surgery, prolonged mechanical ventilation, and prolonged ICU stay (25). These previous studies suggest that preoperative inflammatory CRP level, ESR, and NLR can potentially aid clinicians in risk stratification of patients undergoing cardiovascular surgery. Based on our results, it is suggested that these preoperative indicators may also serve as predictors of poor outcomes after heart valve surgery.

As the optimized cut-off value for the prediction of different outcomes was unclear, this study chose to use ROC analysis to determine such value. In the second part of the study, we confirmed through ROC curves that CRP was a better predictor of early postoperative cardiac insufficiency outcomes (BNP level >400 pg/ml) than ESR and NLR, and we calculated an optimal CRP cut-off point of 5 mg/L. NLR was a better predictor of death within 30 days after surgery than CRP levels and ESR, and the optimal NLR cut-off point was 3.5. Multivariate logistic regression analysis adjusted for demographic data, preoperative complications, and intraoperative data showed that CRP level >5 mg/L was an independent predictor of postoperative cardiac dysfunction, and NLR >3.5 was an independent predictor of death within 30 days after surgery. In a previous prospective observational study, CRP level >3 mg/L was significantly associated with in-hospital death after coronary artery bypass grafting (26). However, the association between preoperative CRP level and early postoperative cardiac insufficiency remains unknown. Based on this study, we confirmed this association and determined a cut-off value for use as a clinical reference.

In recent years, various cardiac function parameters such as the left ventricular stroke volume index or the ejection fraction have been studied to predict postoperative mortality rates (27, 28). However, not all patients are examined for these indicators, and different hospitals may have different standards depending on the equipment and/or operator. Conversely, blood biochemical indicators are more objective, and inflammatory parameters were explored in this study. Regarding the NLR, a retrospective study of aortic valve replacement for aortic stenosis reported that NLR ≥3 was an independent predictor of short- and long-term mortality (29). However, the latter study only included 234 patients, while the present study included more than 3000 patients as well as more confounding factors in the regression model. Based on the current study, we determined NLR >3.5 as the optimal cut-off value for prediction of death within 30 days after surgery. Thus, we believe that this study’s determined significant prognostic factors and diagnostic threshold values for patients undergoing heart valve surgery will provide useful references for the clinical identification of high-risk patients and the formulation of targeted diagnosis and treatment strategies.

The results of this study should be interpreted with the following limitations in mind. First, this was a single-center retrospective cohort study, which makes it difficult to avoid the inherent limitations associated with retrospective studies. Further prospective studies are needed to verify these results in the future. Second, the results of this study are limited to patients undergoing open-heart valve surgery during CPB; whether this conclusion applies to patients undergoing other surgeries needs to be further analyzed and studied in the future. In addition, we focused on the parameter with the maximum AUC to explore its role in postoperative outcomes, and we did not present other parameters with statistical significance. Therefore, the direct effects of other parameters on different postoperative outcomes require further validation.

In summary, this retrospective study showed that the CRP level, ESR, and NLR had a certain predictive effect on postoperative adverse outcomes in patients undergoing heart valve surgery. Thus, it is recommended to administer preoperative anti-inflammatory compounds to reduce the inflammatory load and decrease the potential risk of adverse events in these patients. CRP level >5 mg/L and NLR >3.5 showed relatively high predictive power in terms of postoperative cardiac insufficiency and death within 30 days after surgery, respectively. Preoperative CRP levels and NLR deserve further consideration during preoperative evaluation, and the corresponding adverse events should be given special attention. However, prospective studies are necessary to validate the current findings in the future.

## Data availability statement

The raw data supporting the conclusion of this article will be made available by the authors, without undue reservation.

## Author contributions

HT, XJ, XB, and HH contributed to study design. HT, XJ, QL, XB, GD, JC, SL, and YZ contributed to data collection. HT, GD, and XJ contributed to data analysis. XB, HT, XJ, and HH contributed to writing the manuscript. All authors contributed to the article and approved the submitted version.
